# A Novel Monitoring Navigation Method for Cold Atom Interference Gyroscope

**DOI:** 10.3390/s19020222

**Published:** 2019-01-09

**Authors:** Lin Zhang, Wei Gao, Qian Li, Runbing Li, Zhanwei Yao, Sibin Lu

**Affiliations:** 1 Institute of Navigation Instrument, Harbin Institute of Technology, Harbin 150001, China; gaow@hit.edu.cn; 2 College of Automation, Harbin Engineering University, Harbin 150001, China; 3 State Key Laboratory of Magnetic Resonance and Atomic and Molecular Physics, Wuhan Institute of Physics and Mathematics, Chinese Academy of Sciences, Wuhan 430071, China; rbli@wipm.ac.cn (R.L.); yaozhw@wipm.ac.cn (Z.Y.); lusibin@wipm.ac.cn (S.L.)

**Keywords:** cold atom interference gyroscope, Kalman filtering, monitoring navigation system

## Abstract

The implementation principle of a typical three-pulse cold atom interference gyroscope is introduced in this paper. Based on its configuration and current research status, the problems of cold atom interference gyro are pointed out. The data-rate is insufficient, and it is difficult to achieve high dynamic measurement. Then, based on these two limitations, a novel design of the monitoring navigation system of the cold atom interference gyroscope (CAIG) and an intermediate-grade inertial measurement unit (IMU) was proposed to obtain the long-term position result without GPS signals, such as the Inertial Navigation System (INS) in underwater vehicles. While the CAIG was used as the external gyro, the bias of IMU and the misalignment angle between the CAIG-frame and the IMU-frame are obtained through filtering technique. The simulation test and field test demonstrated the improvements of the long-term positioning accuracy of the INS.

## 1. Introduction

Due to its advantages of complete autonomy, undisturbed, large-amount of output information, and robust real-time performance, the Inertial Navigation System (INS) has irreplaceable benefits in both military and civilian fields, and the application requirements continue to promote the development of inertial sensors. Since the accuracy of gyroscopes and accelerometers directly affects the accuracy of positioning and attitude output of Strapdown Inertial Navigation Systems (SINSs) [1,2,3,4], the development of inertial navigation technology can approximately rely on the advance of two inertial sensor technologies, especially for the gyroscope.

The first-generation gyroscope is represented by electrostatic rate gyro and mechanical gyro [5,6,7]. At present, the bias stability of mechanical gyros is better than $10^{-4}$ ${(}^{\circ }$/h). The second-generation gyroscope is represented by the ring-laser gyroscope and the fiber-optic gyroscope (FOG), and the bias stability is better than $10^{-3}$ ${(}^{\circ }/$h) [8,9]. As a representative of the newest generation of gyro, the cold atom interference gyroscope is based on the Sagnac effect of the atomic de Broglie wave, and its theoretical precision may reach $10^{-12}$ ${(}^{\circ }/$h) [10]. Even though the current cold atomic gyro has many problems to be resolved from practical engineering applications, it has significant precision merits and considerable application prospects.

Since the first implementation of the atom-based interferometer at Stanford University in 1991 [11], atomic cooling and steering technology has developed rapidly. At present, with the atomic excitation, capture, manipulation, and the continuous reduction of the limit temperature of the atom cloud, atomic material wave related technologies have received extensive attention, and successively realized new prototypes such as atomic fountain clocks [12,13,14,15,16] and atomic gravimeters [17,18,19,20,21], have both surpassed the precision of traditional ones. It is worth noting that, due to the technology limitations, cold atom interferometers are mainly used for constant measurements, such as gravitation constants, local gravity, and measurement of basic physical constants [22,23,24,25,26]. Based on the measurement principle of CAIG, which is explained in Section 2, its dynamic measurement range is insufficient to perform a dynamic navigation test. In particular, the data-rate of CAIG cannot realize an independent inertial navigation system, and most of them are used to measure the earth’s rotation constant [10,27,28,29,30].

As the newest-generation sensor for rotation measurement, the CAIG with ultra-high precision potential leads to a breakthrough of IMUs, at the same time, it brings opportunities to solve long-term positioning problem of underwater vehicles without GPS signals. The CAIG‘s advantage lies in long-term stability, repeatability, and extremely high expected sensitivity. Unfortunately, its data-rate is too low to compose an INS till now. This insufficient data-rate will lead to algorithmic solution error. At the same time, the limited dynamic range of the CAIG cannot meet the requirement of accurately capturing the dynamic information of the carrier [31,32]. On the other hand, FOG is an advanced inertial sensor for rotation measurement of carriers. It obtains the advantages of high data-rate and large dynamic range. However, due to the limited improvement, the FOG cannot satisfy the long-term accuracy requirements in terms of bias stability and repeatability. To the best of our knowledge, there are no studies about combining CAIG and FOG. In this paper, a novel monitoring navigation method for CAIG is proposed, and dedicated to combining the benefits of the CAIG and FOG to improve the positioning accuracy of the SINS. When the underwater vehicle is in a lower dynamic environment, the CAIG is used to estimate the bias of the FOG. Otherwise, a mature SINS of FOG is used for navigation in a highly dynamic environment. This monitoring navigation method with lower requirements of CAIG’s data rate, and the CAIG’s measurement accuracy is fully utilized under the limited dynamic range.

In Section 2, the configuration of the CAIG is introduced, and the reasons for the lack of dynamic measurement and the data rate are explained. In Section 3, the design of the monitoring navigation system is introduced, and simulation analysis in Section 4 shows the conditions to realize a fast and accurate monitoring system. Section 5 shows the results of land vehicle experiments and ship experiments, with a significant improvement of the positioning accuracy. In order to reduce the amount of calculation and visually show the effect of this monitoring system [33,34], the classic Kalman filter is chosen to obtain the optimal solution of the time-invariant linear system.

## 2. Three-Pulse Cold Atom Interferometer for Rotational Measurement

**Figure 1:**
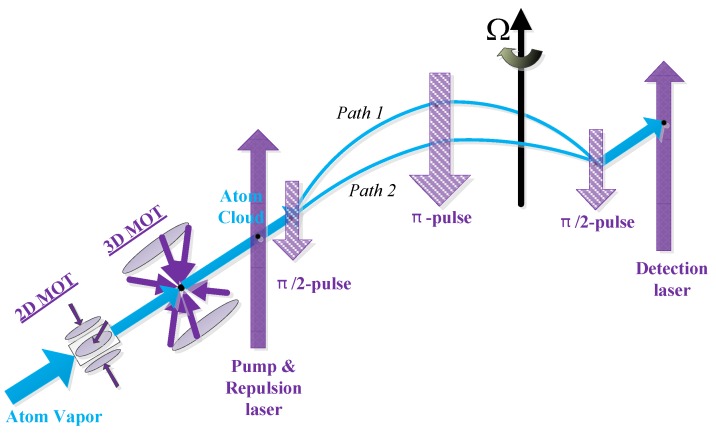
System implementation of cold atom interference gyroscope.

The configuration of the rotation measurement based on the cold atom interferometer mainly includes three-pulse design and four-pulse design. In order to easily realize the triaxial measurement, the three-pulse opposite projection configuration is selected as an example in this paper. As shown in Figure 1, the implementation of CAIG is mainly divided into atom cloud preparation, cooling, state selection, and velocity selection process, interference process, and final atomic state detection. Among them, the imprisonment and manipulation of the atom cloud are all realized by laser control technique, which requires a precise optical system for frequency, phase and light emphasizing. In order to achieve better measurement accuracy and reduce the influence of the external environment, we hope that the external environment of the atomic interference gyroscope achieves vacuum, low temperature, magnetic shielding, and vibration isolation.

### 2.1. Atomic Cooling and Trapping Progress

In order to reduce the interference errors caused by the atom position error, velocity distribution and other factors to the interference result, the atom in the initial state of steam needs to be cooled to the extremely low temperature of the μK-level and trapped as an easy-to-manipulate atom cloud [35,36,37]. The existence time is prolonged through this progress.

When a precise laser illuminates an atom, as long as the oscillation frequency of the atom is equal to the frequency of the laser, the atom absorbs photons and undergoes a transition to reach an excited state. Depending on the principle of energy conservation, the momentum of the atom itself is greatly reduced. On the other hand, the atomic state in the excited state is extremely difficult to maintain, then it will also spontaneously radiate out photons. These photons are emitted to all directions. Accompanied by photon emission, the momentum of the atoms by further losses. In short, atomic cooling is the process by which the laser’s constant action on the atom slows it down. In the same way, we can achieve precise control of the velocity and direction of atoms through precise control of the laser, so-called the atomic manipulation [38].

In order to obtain a better signal-to-noise ratio, we should prepare the atom cloud to reach the magnitude of $10^{8}$ to $10^{9}$ atoms. As shown in Figure 1, ${}^{87}Rb$ vapor becomes an atomic cloud through the action of 2D-MOT and 3D-MOT. After the capture zone, the number of atom clouds is continuously reduced due to factors such as the diffusion of cold atom clouds and the collision of various cavity walls. Furthermore, when there is background atom gas in the vapor chamber, the probe light will emit a large amount of stray fluorescence, which will increase the background noise and decrease the signal-to-noise ratio.

In short, considering the importance of atom cloud preparation and its requirements, the atom cloud preparation process with high reliability is an important guarantee for achieving high precision of the atomic gyro. This is the reason why we use more time to get more atoms into the interference area, eventually led to a lower data rate of CAIG.

### 2.2. Atomic State-Selection Progress

In the process of atomic interference, the interference is usually selected to the D2 line of ${}^{87}Rb$, so that the ${}^{87}Rb$ atom enters the specific cycle between the ground state and the excited state, and gives the rotation information according to the population of atoms in each energy state. To avoid the effects of stray atoms and to improve the signal-to-noise ratio, the progress of atomic state-selection is necessary [39].

**Figure 2:**
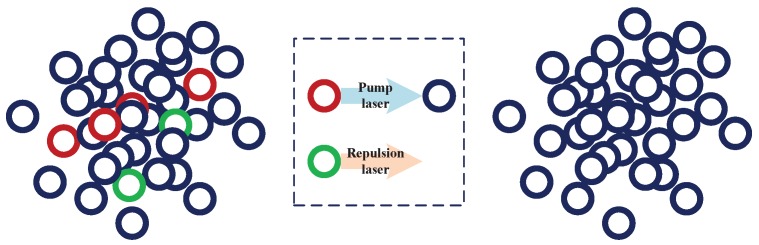
Atomic state-selection Technique.

Depending on the complex energy state structure of ${}^{87}Rb$, atoms at each state can only absorb the specific frequency and then transit to a excited state. The selected interference progress is between $|5^{2}S_{1/2},F=1\rangle \to |5^{2}S_{1/2},F=2\rangle$. Before the interference progress, the pump laser is used to transfer the available atoms of other energy levels $|5^{2}S_{1/2},F=1\rangle$, as is shown in Figure 2.

In addition, there are other $|m_{F}\neq 0\rangle$ atoms that will reduce the signal-to-noise ratio of the interference result due to changes in their sensitive magnetic fields. They are generally blown away by repulsion laser, and finally a pure atom cloud is obtained and trapped in a three-dimensional magneto-optical trap, and then they are ready to enter the interference region.

### 2.3. Atomic Interference Progress

In this section, a three-pulse interferometer is taken as an example to introduce the interference progress of CAIG. When the action laser is detuned to $\delta_{12}-\delta_{AC}=0$, the most simplified transition probability with the Raman frequency $\Omega_{r}=\Omega_{eff}$ can be obtained by

(1) $$\begin{array}{ll} P_{g\to e} & =\sin^{2}\frac{\Omega_{eff}t}{2}\\ & =\frac{1}{2}\left(1-\cos\Omega_{eff}t\right) \end{array}$$

It can be seen that the transition probability fluctuates with the laser duration *t*. When the duration of the pulse is $t=\pi /\left(2\Omega_{r}\right)$, then half of the atoms will undergo state transition, and this pulse is called ‘π/2-pulse’. The ‘π/2-pulse’ is used as a splitter mirror in the optical interferometry. On the other hand, When the duration of the pulse is $t=\pi /\Omega_{r}$, then all of the atoms will undergo state transition, and this pulse is called ‘π-pulse’. The ‘π-pulse’ is used as a reflecting mirror in the optical interferometry. In the atomic interference region, three pairs of Raman laser are used as a splitter mirror, reflect mirror, and splitter mirror in the optical interferometry. In summary, we can control the atom cloud by controlling the Raman pulses to realize the atomic interferometer shown in Figure 1.

Interferometric gyroscopes [40], including optical interference gyros and cold atomic interference gyros, are based on the Sagnac effect, as shown in (2). The core issue of interferometric gyroscopes is to measure and separate the phase shift caused by the rotation.

(2) $$\Phi_{Sagnac}=\frac{4\pi E}{hc^{2}}A\cdot \Omega$$

**Figure 3:**
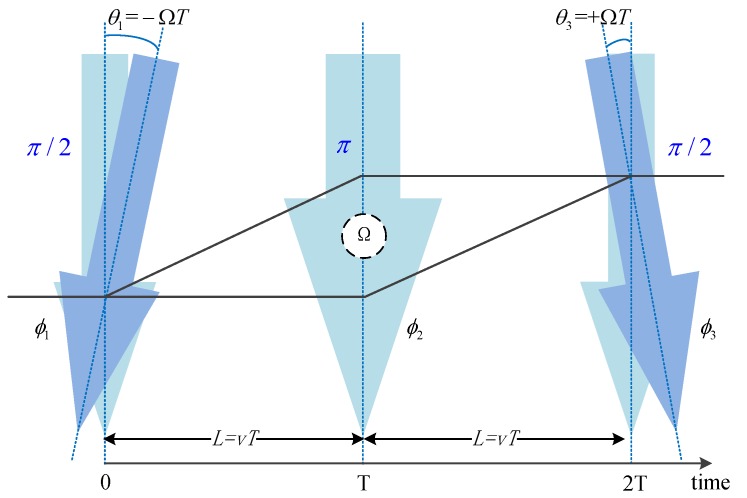
Schematic diagram of the Doppler effect.

As shown in Figure 3, the phase shift caused by the rotation caused by the Doppler effect is

(3) $$\Phi_{Doppler}=\left(\phi_{1}-\phi_{2}^{A}\right)-\left(\phi_{2}^{B}-\phi_{3}\right)$$

where $\phi_{i}$ is the phase from the ith pulse, $\phi_{i}={k_{eff}}^{\left(i\right)}\cdot z\left(t\right)+\phi_{arb}^{\left(i\right)}$, related to the wave vector $k_{eff}$, the atomic position z(t), and the random phase of the pulse $\phi_{arb}$. Due to the Doppler effect, a slight deflection θ of the pulse occurs when the pulse acts on the atom, θ=±ΩT≪1. When we ignore random phases, there are

(4) $$\left\{\begin{array}{c} \phi_{1}\approx k_{eff}\cdot \theta_{1}L=-k_{eff}\cdot \Omega T\cdot L\\ \phi_{2}^{A}=-\phi_{2}^{B}\\ \phi_{3}\approx -k_{eff}\cdot \theta_{3}\cdot L=-k_{eff}\cdot \Omega T\cdot L \end{array}\right.$$

Substituting (4) into (3) results in a phase shift $\Phi_{Doppler}$,

(5) $$\begin{array}{ll} \Phi_{Doppler} & =\left(\phi_{1}-\phi_{2}^{A}\right)-\left(\phi_{2}^{B}-\phi_{3}\right)\\ & =-2k_{eff}\cdot \Omega \cdot TL=-2k_{eff}\cdot \Omega T\cdot v\cdot T \end{array}$$

When the actual rotation axis is not perpendicular to the interference plane, (5) is corrected into (6). Defining equivalent interference area $A=-\left(\hbar k_{eff}/M\right)T\times vT=-\left(\hbar k_{eff}/M\right)T\times L$, (5) is converted into $\Phi_{Doppler}=2MA\cdot \Omega /\hbar$. Therefore, the Doppler effect phase shift caused by rotation is equal to the Sagnac phase expression of (2), where *ℏ* is the reduced Planck constant. On the other hand, it can be seen that the interference area *A* is proportional to the square of the pulse interval time *T*, that is how the time domain atomic gyro increases its sensitivity.

(6) $$\Phi_{Doppler}=-2k_{eff}\cdot \left(\Omega \times v\right)T^{2}$$

Based on the above principle of rotation measurement, the output phase of the atomic gyro in the laboratory consists of several parts, as shown in (7).

(7) $$\Delta \Phi_{total}=k_{eff}\cdot g\cdot T^{2}+\frac{2MA}{\hbar }\Omega -2k_{eff}\cdot \left(\Omega \times g\right)T^{3}+\Delta \Phi^{0}$$

where $k_{eff}\cdot g\cdot T^{2}$ is the phase part caused by gravity, 2MAΩ/ℏ is the phase part caused by rotation, $-2k_{eff}\cdot \left(\Omega \times g\right)T^{3}$ is the phase correction when gravity g is not parallel to the actual rotation axis, and $\Phi_{arb}$ is the Random phase introduced by three pulses, $\Phi_{arb}=\phi_{arb}^{\left(1\right)}-2\phi_{arb}^{\left(2\right)}+\phi_{arb}^{\left(3\right)}$.

**Figure 4:**
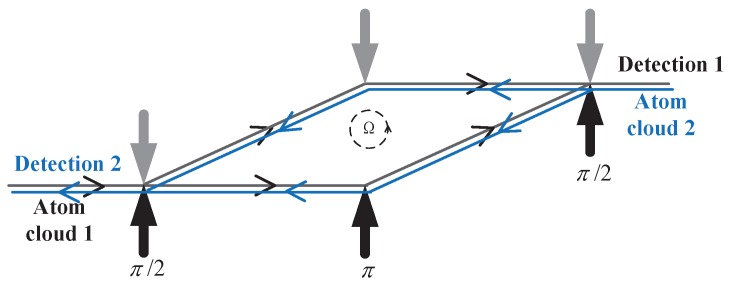
Dual interference loops with opposite-projectile atom clouds.

Typical dual interference loops with opposite-projectile atom clouds are shown in Figure 4, and we can get a phase shift from each loop. As shown in (8), we can process the two-loop data output to obtain the common mode part and the differential mode part.

(8) $$\left\{\begin{array}{cc} \Phi_{tot1} & =k_{eff}\cdot g\cdot T^{2}+\frac{2m}{\hbar }\Omega \cdot A_{1}-2k_{eff}\cdot \left(\Omega \times g\right)T^{3}+\Delta \Phi^{0}\\ \Phi_{tot2} & =k_{eff}\cdot g\cdot T^{2}+\frac{2m}{\hbar }\Omega \cdot A_{2}-2k_{eff}\cdot \left(\Omega \times g\right)T^{3}+\Delta \Phi^{0} \end{array}\right.$$

In two interference loops, the equivalent loop area vector $A_{1}=-A_{2}$. Then the phase shift related to the rotation is successfully extracted by

(9) $$\Phi_{tot1}-\Phi_{tot2}=\frac{2m}{\hbar }\Omega \cdot \left(A_{1}-A_{2}\right)=\frac{4m}{\hbar }\Omega \cdot A_{1}$$

### 2.4. Fluorescence Detection Progress

After the interference process, the population of the atomic state is obtained by fluorescence detection progress [41]. The ratio of atoms in two ground state $|5^{2}S_{1/2},F=1\rangle$ and $|5^{2}S_{1/2},F=2\rangle$ is calculated and converted into the transition probability result of the interference process.

(10) $$P_{a\to b}=|C_{b}\left(t\right){|}^{2}=\frac{1}{2}\left(1-\cos\Delta \Phi \right)$$

The absorption imaging technique is adopted here, and the fluorescence multiplier is received by the photomultiplier tube, and the total number of atoms at different energy states is obtained by time domain integration, in another word, the ratio of the population is obtained. In the specific implementation process, it is necessary to detect the laser light intensity and frequency to obtain precise control, and also needs to isolate stray light isolation, external temperature, and electromagnetic field isolation treatment.

It is worth noting that during the probing process, we need a unique and determined 2π period from the conversion of the ratio of the population $P_{a\to b}$ to the phase result ΔΦ. In order to achieve dynamic measurements, it is common to reduce the scale factor to obtain a larger range of interference phase result which is from larger angular velocity variations. In order to maintain the system accuracy of the cold atom interference gyroscope, only a small dynamic measurement range can be obtained, and it still cannot be independently used for the dynamic measurement.

## 3. The Novel Monitoring Navigation Method of Triaxial CAIG & FOG

The principled shortcomings of CAIG have fully explained above. In this section, a new scheme is proposed to improve the positioning accuracy of the conventional SINS through additional CAIG. Here we take FOG as an example, which is most commonly used in underwater vehicles.

### 3.1. The Monitoring Navigation Scheme of Triaxial CAIG & FOG

**Figure 5:**
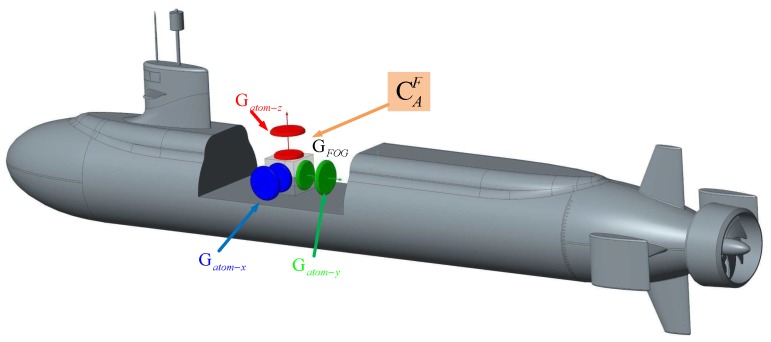
The monitoring navigation configuration scheme of triaxial cold atom interference gyroscope (CAIG) & fiber-optic gyroscope (FOG).

As is known that bias drift of FOG is the main error source affecting the accuracy of SINSs. In the monitoring navigation system, the triaxial cold-atom interference gyroscope is used as an additional gyro to monitor an IMU of FOGs, as is shown in Figure 5, the triaxial FOG and CAIG is fixed to the underwater vehicle with the same body-frame. It is worth noting that we expect the measurement axes of FOG and CAIG to be approximately parallel to ensure the linearity of the system. The difference between the triaxial rotation rate of the CAIG and the FOG is the measurement observations. Through the Kalman filtering, the triaxial bias of the FOG is calculated and corrected, so that the monitoring navigation system works in the closed loop state. Due to the limitation of CAIG in dynamic measurement, the monitoring system achieves the estimation of the gyro through CAIG in a relatively stable process. In the case of higher dynamics, the navigation output of position and attitude are performed by the fiber-optic SINS. In order to reduce the influence of data out-of-synchronization on the performance of the monitoring system, the rotation rate output of the cold atom interference gyroscope and the fiber-optic gyroscope needs to be synchronized by data processing to realize the estimation of the gyro drift. Finally, the positioning accuracy of the long-term navigation based on the fiber-optic SINS can be improved.

In practical engineering, an inevitable misalignment angle between the cold atom interference gyroscope frame (represented by A-frame) and the fiber-optic gyroscope frame (represented by F-frame) exists. The existence of the misalignment angle will directly affect the estimation of the triaxial gyro drift. Considering the constant misalignment angle, the direction cosine matrix $C_{A}^{F}$ between A-frame and F-frame

(11) $$C_{A}^{F}=I-\left[\varphi_{a}\times \right]$$

where, $\left[\varphi_{a}\times \right]$ is the anti-symmetric matrix of misalignment angle $\varphi_{a}={\left[\varphi_{ax},\varphi_{ay},\varphi_{az}\right]}^{T}$.

The rotation rate $\omega_{ib_{A}}^{b_{A}}$ measured by CAIG is transformed into the F-frame from A-frame,

(12) $$\omega_{ib_{F}}^{b_{F}}=C_{A}^{F}\omega_{ib_{A}}^{b_{A}}$$

As is known, the measured values of gyros are

(13) $$\left\{\begin{array}{l} {\hat{\omega }}_{ib_{F}}^{b_{F}}=\omega_{ib_{F}}^{b_{F}}+\varepsilon_{F}\\ {\hat{\omega }}_{ib_{A}}^{b_{A}}=\omega_{ib_{A}}^{b_{A}}+\varepsilon_{A} \end{array}\right.$$

where, ${\hat{\omega }}_{ib_{F}}^{b_{F}}$ and ${\hat{\omega }}_{ib_{A}}^{b_{A}}$ are the measured value of rotation rate of FOG and CAIG, $\omega_{ib_{F}}^{b_{F}}$ and $\omega_{ib_{A}}^{b_{A}}$ are the actual value of rotation rate in F-frame and in A-frame. $\varepsilon_{F}$ is the bias of FOG, $\varepsilon_{F}={\left[\varepsilon_{Fx},\varepsilon_{Fy},\varepsilon_{Fz}\right]}^{T}$, and $\varepsilon_{A}$ is the bias of CAIG, $\varepsilon_{A}={\left[\varepsilon_{Ax},\varepsilon_{Ay},\varepsilon_{Az}\right]}^{T}$.

In the triaxial CAIG & FOG monitoring navigation system, the misalignment angle $\varphi_{a}$, and the bias of FOG $\varepsilon_{F}$ is constant, then the system state equation is established as (14),

(14) $$X_{k+1}=\Phi_{k+1,k}X_{k}+\Gamma_{k}W_{k}$$

where, the state variables $X={\left[\varphi_{ax},\varphi_{ay},\varphi_{az},\varepsilon_{Fx},\varepsilon_{Fy},\varepsilon_{Fz}\right]}^{T}$.

And the observation is the rotation rate difference δω between the CAIG & FOG,

(15) $$\begin{array}{ll} \delta \omega & ={\hat{\omega }}_{ib_{F}}^{b_{F}}-{\hat{\omega }}_{ib_{A}}^{b_{A}}\\ & =\varepsilon_{F}-\varepsilon_{A}+\omega_{ib_{A}}^{b_{A}}\left[\varphi_{a}\times \right] \end{array}$$

where, the bias of high-precision cold atom interference gyroscope $\varepsilon_{A}$ is much smaller than $\varepsilon_{F}$, and can be ignored here. Then (15) can be simplified to

(16) $$\delta \omega =\omega_{ib_{A}}^{b_{A}}\left[\varphi_{a}\times \right]+\varepsilon_{F}$$

Organize (16) into a matrix form, the observation equation is established as

(17) $$Z_{k}=H_{k}X_{k}+V_{k}$$

where $V_{k}$ is a zero mean valued Gaussian white noises with variance of $R_{k}$, and the observing matrix is

(18) $$H_{k}=\left[\begin{array}{cccccc} 0 & -\omega_{ib_{Az}}^{b_{A}} & \omega_{ib_{Ay}}^{b_{A}} & 1 & 0 & 0\\ \omega_{ib_{Az}}^{b_{A}} & 0 & -\omega_{ib_{Ax}}^{b_{A}} & 0 & 1 & 0\\ -\omega_{ib_{Ay}}^{b_{A}} & \omega_{ib_{Ax}}^{b_{A}} & 0 & 0 & 0 & 1 \end{array}\right]$$

### 3.2. The Observability Analysis of Monitoring Navigation System

We gave the model of the monitoring system in the previous section, and then the next step is to perform an observability analysis. One of the reasons for observability analysis is the need to determine the efficiency of the Kalman filter designed to estimate the state of the system. On the other hand, if the observability of the system is poor, we cannot obtain an accurate estimate of the state even if the noise level is negligible. In other words, the observability analysis determines the conditions under which the system can obtain an accurate estimate of the state.

In observability analysis theory, the system of interest is the homogeneous system. Consider the observation equation of a continuous system

(19) $$z_{j}\left(t\right)=H_{j}x(t)$$

where, $z_{j}\left(t\right)$ is the observation in *j*-th period, and $H_{j}$ is the observing matrix.

Differentiate the measurement vector n-1 times inside each segment to obtain

(20) $$\left\{\begin{array}{l} z_{j}\left(t\right)=H_{j}x\left(t\right)\\ {\dot{z}}_{j}\left(t\right)=H_{j}\dot{x}\left(t\right)=H_{j}\Phi_{j}x\left(t\right)\\ {\ddot{z}}_{j}\left(t\right)=H_{j}\ddot{x}\left(t\right)=H_{j}{\Phi_{j}}^{2}x\left(t\right)\\ \vdots \\ z_{j}^{\left(n-1\right)}\left(t\right)=H_{j}x^{\left(n-1\right)}\left(t\right)=H_{j}{\Phi_{j}}^{n-1}x\left(t\right) \end{array}\right.$$

which can be written as

(21) $$Z_{j}\left(t\right)=Q_{j}X\left(t\right)$$

where, $Q_{j}$ is the observability matrix in the *j*-th period,

(22) $${Q_{j}}^{T}=\left[\begin{array}{ccccc} {H_{j}}^{T} & {\left(H_{j}\Phi_{j}\right)}^{T} & {\left(H_{j}\Phi_{j}^{2}\right)}^{T} & \cdots & {\left(H_{j}\Phi_{j}^{n-1}\right)}^{T} \end{array}\right]$$

According to the principle of piece-wise constant systems (PWCS) observability analysis theory [42], when the rank of the Total Observability Matrix $Q\left(r\right)$ is equal to the number of observable state variables, the system is fully observable. Furthermore, as the system model shown in Section 3.1, the observability matrix for the *j*-th period can be written as

(23) $$Q\left(r\right)=\left[\begin{array}{c} Q_{1}\\ Q_{2}\Phi^{n-1}\\ \vdots \\ Q_{r}\prod_{1}^{r-1}\Phi^{n-1} \end{array}\right]=\left[\begin{array}{c} Q_{1}\\ Q_{2}\\ \vdots \\ Q_{r} \end{array}\right]$$

In our monitoring system, there is

(24) $${Q_{r}}^{T}=\left[{\left(H_{j}\right)}^{T},{\left(H_{j}\Phi \right)}^{T},\ldots ,{\left(H_{j}\Phi^{n-1}\right)}^{T}\right]=\left[{\left(H_{j}\right)}^{T},{\left(H_{j}\right)}^{T},\ldots ,{\left(H_{j}\right)}^{T}\right]$$

(25) $$H_{j}=\left[\begin{array}{cccccc} 0 & -\omega_{ib_{Az}}^{b_{A}}\left(j\right) & \omega_{ib_{Ay}}^{b_{A}}\left(j\right) & 1 & 0 & 0\\ \omega_{ib_{Az}}^{b_{A}}\left(j\right) & 0 & -\omega_{ib_{Ax}}^{b_{A}}\left(j\right) & 0 & 1 & 0\\ -\omega_{ib_{Ay}}^{b_{A}}\left(j\right) & \omega_{ib_{Ax}}^{b_{A}}\left(j\right) & 0 & 0 & 0 & 1 \end{array}\right]$$

where, $\omega_{ib_{Ax}}^{b_{A}}\left(j\right)$, $\omega_{ib_{Ay}}^{b_{A}}\left(j\right)$, $\omega_{ib_{Az}}^{b_{A}}\left(j\right)$ corresponds to the triaxial measurements of CAIG.

To simplify the analysis, the respective stripped observability matrix (SOM) $U_{s,0}\left(j\right)$ is

(26) $$U_{s,0}\left(j\right)=\left[\begin{array}{c} \begin{array}{cccccc} 0 & -\omega_{ib_{Az}}^{b_{A}}\left(i\right) & \omega_{ib_{Ay}}^{b_{A}}\left(i\right) & 1 & 0 & 0\\ \omega_{ib_{Az}}^{b_{A}}\left(i\right) & 0 & -\omega_{ib_{Ax}}^{b_{A}}\left(i\right) & 0 & 1 & 0\\ -\omega_{ib_{Ay}}^{b_{A}}\left(i\right) & \omega_{ib_{Ax}}^{b_{A}}\left(i\right) & 0 & 0 & 0 & 1 \end{array}\\ \begin{array}{cccccc} 0 & -\omega_{ib_{Az}}^{b_{A}}\left(j\right) & \omega_{ib_{Ay}}^{b_{A}}\left(j\right) & 1 & 0 & 0\\ \omega_{ib_{Az}}^{b_{A}}\left(j\right) & 0 & -\omega_{ib_{Ax}}^{b_{A}}\left(j\right) & 0 & 1 & 0\\ -\omega_{ib_{Ay}}^{b_{A}}\left(j\right) & \omega_{ib_{Ax}}^{b_{A}}\left(j\right) & 0 & 0 & 0 & 1 \end{array} \end{array}\right]$$

Therefore, if there are any two time periods *i*, *j*, (27) is established, $rank[U_{s,0}\left(j\right)]\;=\;6$, and the system’s six state variables are completely observable.

(27) $$\left\{\begin{array}{l} \omega_{ib_{Ax}}^{b_{A}}\left(i\right)-\omega_{ib_{Ax}}^{b_{A}}\left(j\right)\neq 0\\ \omega_{ib_{Ay}}^{b_{A}}\left(i\right)-\omega_{ib_{Ay}}^{b_{A}}\left(j\right)\neq 0\\ \omega_{ib_{Az}}^{b_{A}}\left(i\right)-\omega_{ib_{Az}}^{b_{A}}\left(j\right)\neq 0 \end{array}\right.$$

Based on the above observability analysis, common movement mode of underwater vehicles, such as static mode, triaxial sway mode, constant speed mode, and constant acceleration mode is shown here,

- In the static mode and constant speed mode, $rank[U_{s,0}\left(j\right)]=3$, which means the system isn’t completely observable. It is worth noting that in this monitoring navigation system, the accurate estimation of the triaxial bias and the misalignment angle is indispensable, otherwise, the monitoring system loses its accuracy.
- In constant acceleration mode of the underwater vehicle, the latitude and the height change and affect the measurement of earth’s rotation, but these changes will be submerged in the gyro noise and is difficult to be measured.
- In the case of triaxial sway mode, (27) can be realized in several swing periods. The bias and misalignment angle are completely observable. In fact, underwater surge sway is a necessary condition for our monitoring system to achieve identification. On the other hand, when in land vehicles or other carriers, there may be bumps or other motions similar like swaying, the experiment results will be shown in the field test in Section 5.

## 4. Simulation Experiment of Monitoring Navigation System

The simulation verification for the monitoring navigation system described in the previous section is shown here. The bias of the cold atom interference gyroscope is constant and negligible. The bias of the FOG is $0.1{}^{\circ }/h$, and the triaxial misalignment angles between A-frame and F-frame are $1^{\circ }$, $2^{\circ }$, $3^{\circ }$, the data-rate of CAIG is set to 5 Hz, and the FOG‘s data-rate is 100 Hz. The initial value of the Kalman filtering is selected as follows,

(28) $$\begin{array}{ll} X_{0} & ={\left[\;0,\;0,\;0,\;0,\;0,\;0\;\right]}^{T}\\ P_{0} & =diag\left[\left(1^{\circ }\right){}^{2},\left(2^{\circ }\right){}^{2},\left(3^{\circ }\right){}^{2},\left(0.1{}^{\circ }/h\right){}^{2},\left(0.1{}^{\circ }/h\right){}^{2},\left(0.1{}^{\circ }/h\right){}^{2}\right] \end{array}$$

The simulation tests were carried out in Matlab on a computer with an Intel(R) Core(TM) i7-8500U CPU 3.79 GHz and 20 GB of RAM.

### 4.1. Constant Speed Mode

**Figure 6:**
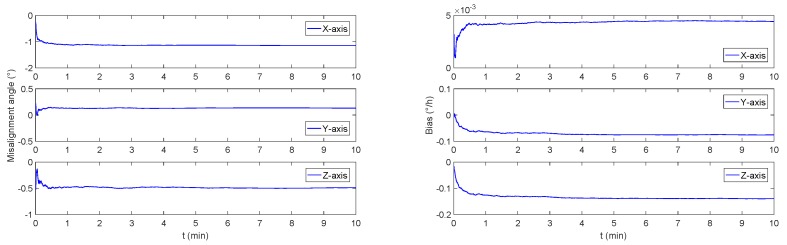
The monitoring system simulation results under constant speed mode. The left figure is the trixial misalignment angle curve, and the right figure is the simulated curve for bias of FOG.

In order to verify the monitoring effect under constant speed motion, the initial heading angle is set to be $30^{\circ }$, both pitch angle and roll angle is set to be $0^{\circ }$, and the underwater vehicle is at the constant speed of 15 knot. As a result of the simulation, the misalignment angle and the gyro bias are shown in Figure 6.

It can be seen that the simulation results are consistent with the conclusions of the observability analysis. The movement of underwater vehicles at the speed of 15 knot cannot achieve effective excitation, and the monitoring system can not estimate the bias of FOG or misalignment angle in a short time.

### 4.2. Constant Accelerate Mode

**Figure 7:**
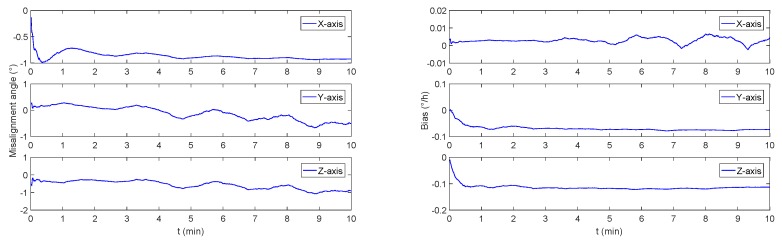
The monitoring system simulation results under constant accelerate mode. The left figure is the trixial misalignment angle curve, and the right figure is the simulated curve for bias of FOG.

In order to verify the monitoring effect under constant accelerate motion, the initial heading angle is set to be $30^{\circ }$, both pitch angle and roll angle is set to be $0^{\circ }$, the initial speed is set to be 0 m/s, and the acceleration is 0.05 $m^{2}/s$. As a result of the simulation, the misalignment angle and the gyro bias are shown in Figure 7.

It can be seen that the stable bias estimation cannot be achieved within ten minutes, which is consistent with the previous analysis.

### 4.3. Triaxial Swing Mode

**Figure 8:**
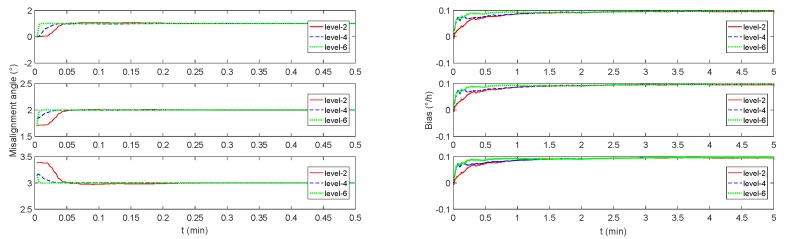
The monitoring system simulation results under triaxial sway mode. The sea condition is level-2, level-4, and level-6, respectively corresponding to the red solid line, blue dashed line, and green dotted line. The left figure is the misalignment angle estimation curve, and the right figure is the simulation estimation curve of the gyro bias.

In order to verify the monitoring effect under triaxial swing mode, the initial heading angle, pitch angle, and roll angle are set to be $0^{\circ }$, the initial speed is set to be 0 m/s, and the acceleration is set to be 0 $m^{2}/s$. As it‘s known that the sea condition is divided into 10 grades referred to Douglas sea scale [43,44], several sway parameters of our ship under typical sea conditions are represented in Table 1. In the sea condition of level-2, the wave is small, the wavelength is short, but the waveform is significant. In the sea condition of level-4, the waves have a distinct shape and form white waves everywhere. In the sea condition of level-6, the wind-cut waves on the crests begin to stretch along the wavy slope into bands. In typical sea conditions, the monitoring navigation system based on CAIG/FOG has been verified. Table 2 is the simulation result and the filter convergence time. At the same time, Figure 8 shows that the misalignment angle can be recognized extremely quickly, and even worse sea condition with more obvious three-axis swing can reduce the convergence time.

The above simulation result shows that the misalignment angle between two frames and the FOG‘s bias are not well estimated in the constant speed mode or in the constant acceleration mode. On the other hand, the triaxial swing mode such as ships in typical sea conditions, the estimation algorithm for the misalignment angle between two frames and the FOG‘s bias is with high precision, especially the convergence time of the estimated misalignment angle is extremely short.

**Figure 9:**
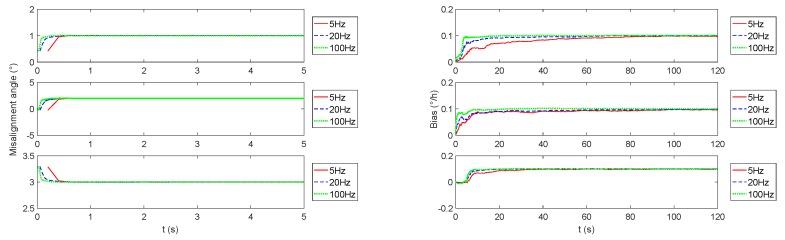
The monitoring system simulation results o uf different dander triaxiata ratel sway mode. The data rate of CAIG is 5 Hz, 20 Hz, and 100 Hz, respectively corresponding to the red solid line, blue dashed line, and green dotted line. The left figure is the misalignment angle estimation curve, and the right figure is the simulation estimation curve of the gyro bias.

Due to the excellent performance of the monitoring navigation system in the case of three-axis sway, we demonstrate the impact of CAIG‘s data rate in Figure 9. It can be seen that the increase in data rate directly corresponds to the decrease of convergence time of the monitoring system, but has no effect on the estimation result. However, in the actual carrier experiment, the difference between the data rates of two gyros may introduce a difference in time label, and then introduce an obvious error into the monitoring navigation system.

## 5. The Field Test of the Monitoring Navigation Method

**Figure 10:**
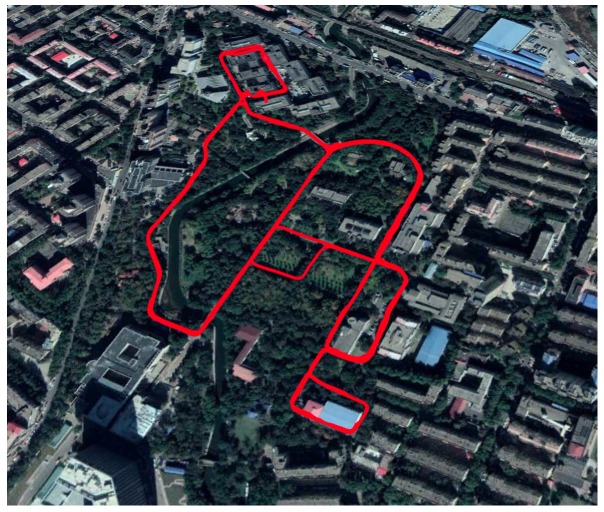
The vehicle experiment route in Harbin Institute of Technology in 4 May 2018.

In this section, we conducted a land vehicle experiment to initially validate the monitoring navigation algorithm. In the field test, there are bumps, direction-shifting, and vehicle steering during the vehicle’s movement. It is expected that gyro’s bias and misalignment angle estimation can be achieved. In 4 May 2018, the vehicle experiment was carried out in the Science Park of Harbin Institute of Technology, as shown in Figure 10.

**Figure 11:**
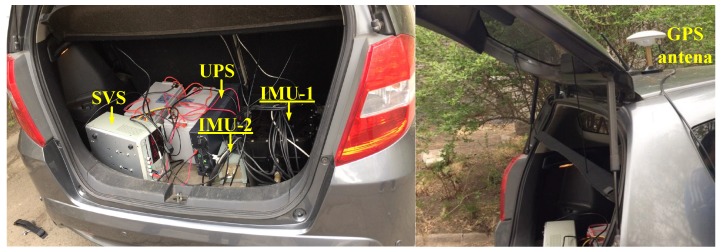
The vehicle experimental device.

**Figure 12:**
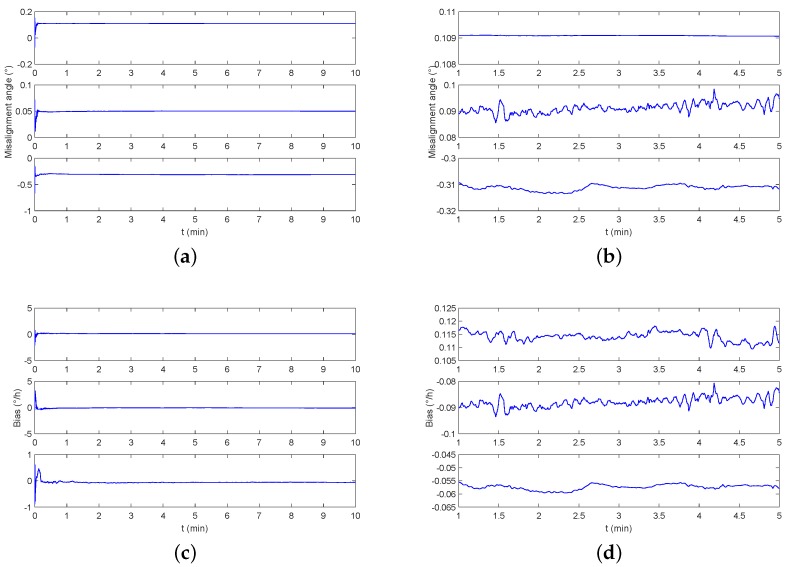
The parameter estimation result of vehicle monitoring experiment. (**a**) The estimation of the misalignment angle after starting the monitoring system. (**b**) The stable estimation details of the misalignment angle. (**c**) The estimation of the triaxial bias after starting the monitoring system. (**d**) The stable estimation details of the triaxial bias.

Figure 11 shows our experimental system, consisting of two INSs, an uninterruptible power supply (UPS), a stabilized voltage supply (SVS), and a GPS receiver. The GPS signal collected here is used to obtain the real position, and to obtain more real speed information as a reference. The results of the following inertial navigation algorithm are not involved in GPS. Limited by the test conditions, two sets of FOGs with different precisions are used for the field experiment. The reference information in the experiment is from a high-precision fiber gyro of the order of 10${}^{\circ }$/h with a data-rate of 100 Hz. The fiber-optic gyroscope being monitored is on the order of 10${}^{\circ }$/h with a data rate of 100 Hz, both gyros are developed by our laboratory. During the experiment, we circled the park six times in one hour, the travel distance was more than 20 km, and the maximum speed of the vehicle exceeded 15 m/s. The monitoring result is shown in Figure 12, it is worth noting that during the experiment, the estimated effect is limited by the dynamic measurement errors of two gyros during the Turns and bumps progress. Referring to the accuracy of the experimental gyro, the estimated value is considered to be stable when the fluctuation of the estimated bias is less than 0.01${}^{\circ }$/h.

**Figure 13:**
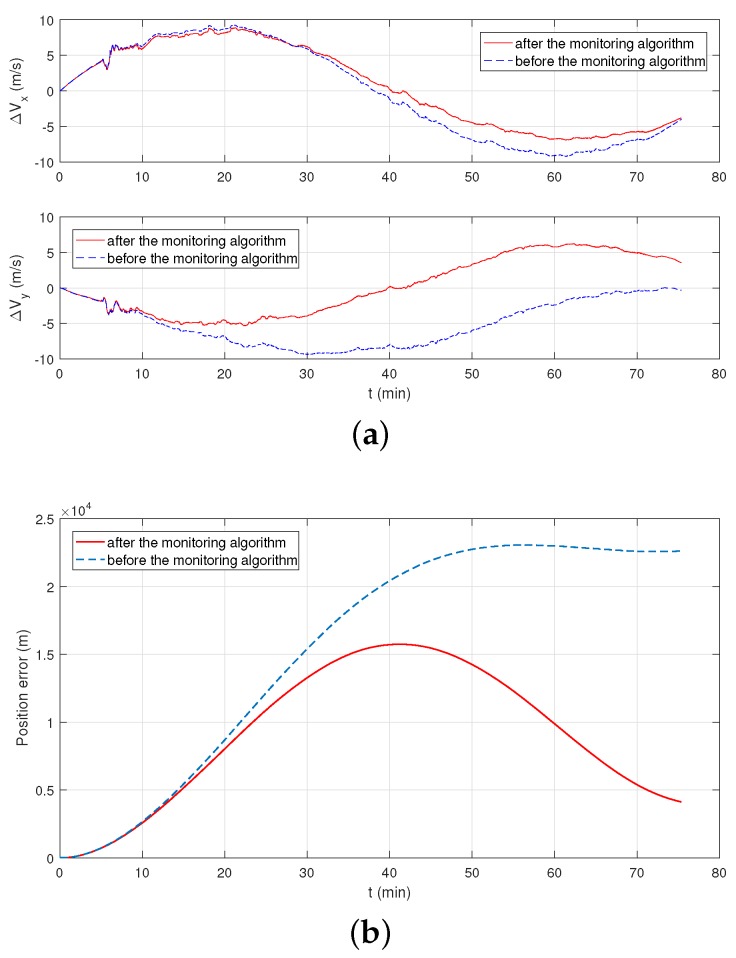
The output result of vehicle monitoring experiment. (**a**) Velocity errors in the east direction and in the north direction. (**b**) Positioning errors of INS.

In Figure 13a, the blue dotted line is the speed error by the normal inertial navigation algorithm without the help of GPS signal, the red solid line is the speed error under the monitoring algorithm. In Figure 13b, the blue dotted line is the positioning error of the original inertial navigation algorithm; the red solid line is the positioning error under the monitoring algorithm. Due to the limitation of the accuracy of the FOG ($10^{-1}{}^{\circ }/h$) in this part, the maximum error of the original inertial navigation system is in line with theoretical expectations. As can be seen from Figure 13, the monitoring system achieves a better speed output and a better positioning result.

**Figure 14:**
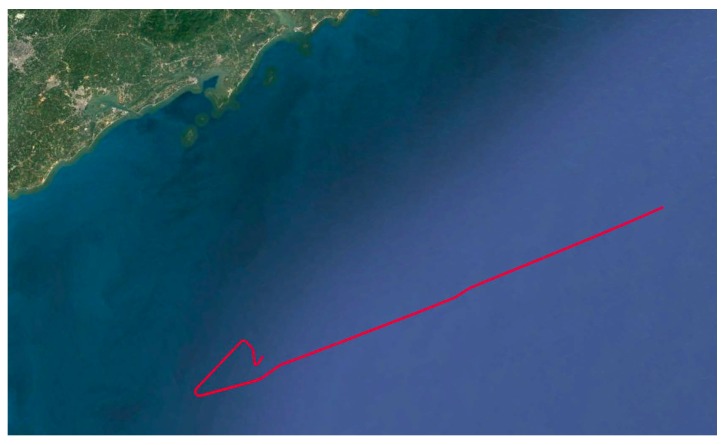
The ship experiment route near Zhanjiang in August 2018.

**Figure 15:**
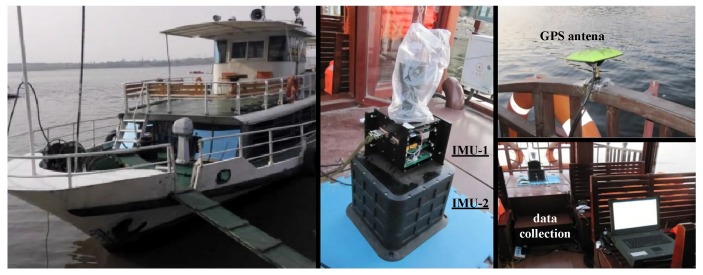
The experimental device of ship carrier.

**Figure 16:**
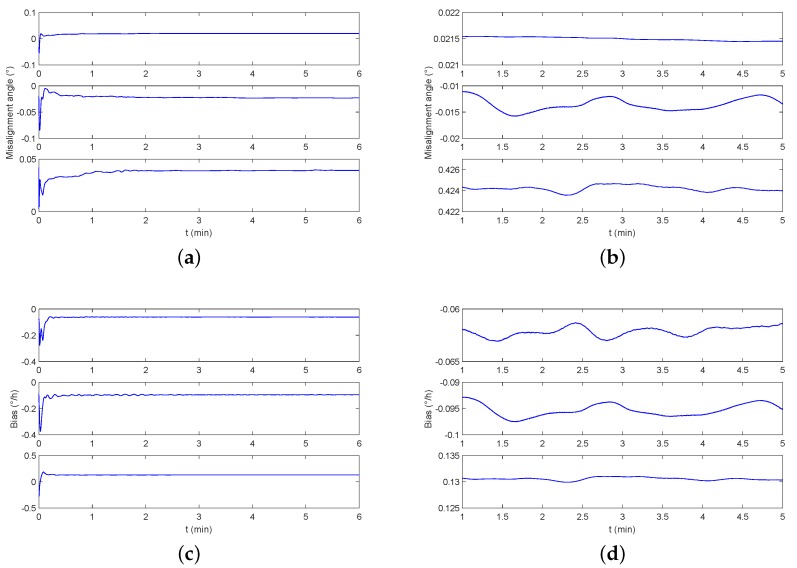
The parameter estimation result of monitoring experiment on level-2 sea condition. (**a**) The estimation of the misalignment angle after starting the monitoring system. (**b**) The stable estimation details of the misalignment angle. (**c**) The estimation of the triaxial bias after starting the monitoring system. (**d**) The stable estimation details of the triaxial bias.

Furthermore, we conducted an experiment in August 2018 in the sea near Zhanjiang, China, and the ship experiment route is shown in Figure 14, while the experimental device is shown in Figure 15, During the experiment, the sea state is about level-2. The reference information in the experiment is from a high-precision gyro of the order of $10^{-3}$ ${}^{\circ }/h$ with a data-rate of 100Hz. The fiber-optic gyroscope being monitored is on the order of $10^{-1}$ ${}^{\circ }/h$ with a data rate of 100 Hz. The monitoring result is shown in Figure 16. It can be seen that a stable estimate of the misalignment angle and the triaxial gyro bias can be achieved at 2 min.

**Figure 17:**
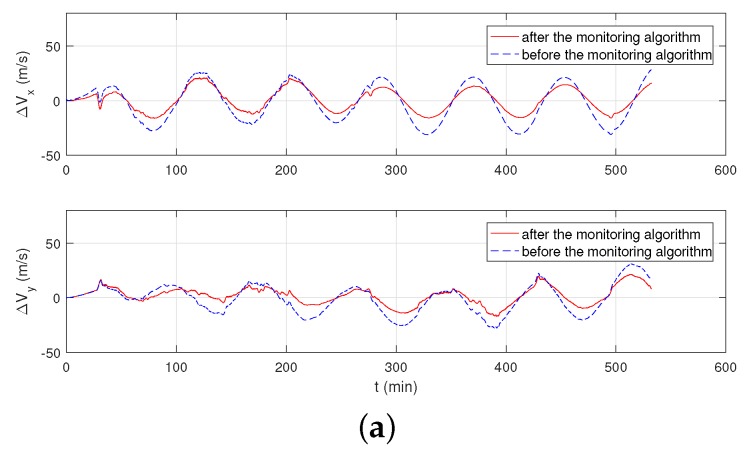

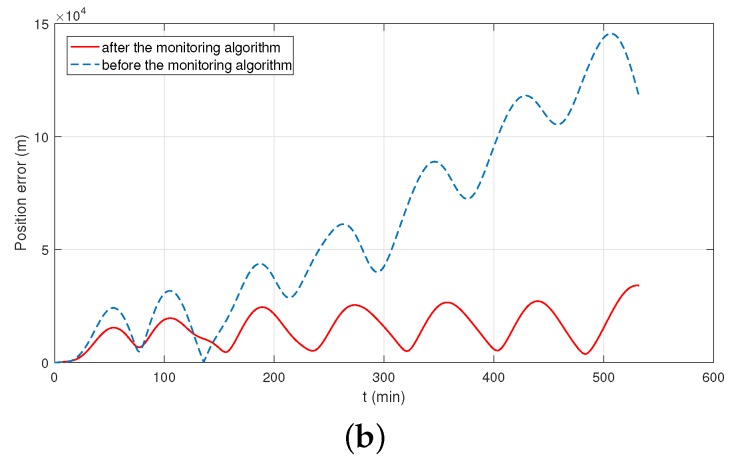
The positioning result of underwater vehicle monitoring experiment. (**a**) Velocity errors in the east direction and in the north direction. (**b**) Positioning errors of INS.

In Figure 17a, the blue dotted line is the speed error by the original inertial navigation algorithm, the red solid line is the speed error under the monitoring algorithm. In Figure 17b, the blue dotted line is the positioning error of the original inertial navigation algorithm; the red solid line is the positioning error under the monitoring algorithm. Due to the limitation of the accuracy of the FOG ($10^{-1}$ ${}^{\circ }/h$) in this part, the maximum error of the original inertial navigation system is in line with theoretical expectations. As can be seen from Figure 17, the monitoring system achieves a better speed output result and a significantly improved positioning result.

## 6. Conclusions

The monitoring navigation system proposed in this paper can achieve an approximate stable estimation of gyro’s bias drift under general dynamic conditions, and compensated to the SINS to obtain a better positioning result. However, due to the defects of CAIG’s dynamic measurement range, it is still necessary to use FOG for navigation in a highly dynamic environment. With the further development of CAIG, the increase in data rate can further accelerate the monitoring estimation progress. Furthermore, a closer combination of FOG and CAIG can be realized, and a rough range of phases can be exactly realized by FOG to achieve high precision under high dynamics measuring.

Considering the current research status of cold atom interference gyroscopes, there are still some limitations in the sea test under this monitoring model. For example, the sampling rate will limit the timing and estimation effect of the position calibration after the monitoring process, and the data delay between the two gyros will directly affect the estimation error. Another important point is that the dynamic performance of the cold atom interference gyroscope is insufficient. Due to CAIG‘s accuracy is proportional to the scale factor, the higher measurement accuracy means a greater possibility that the phase results will appear on other interference stripes, and greatly affecting the measurement performance. 

## Figures and Tables

**Table 1:** The sway parameters of our ship under different sea conditions.

Level	Wave Height (m)	Roll		Pitch		Heading	
		Amplitude	Period	Amplitude	Period	Amplitude	Period
level-2	0.1∼0.5	0.50${}^{\circ }$	20.00 s	0.20${}^{\circ }$	30.00 s	0.30${}^{\circ }$	30.00 s
level-4	1.25∼2.5	4.75${}^{\circ }$	21.00 s	0.64${}^{\circ }$	10.00 s	0.70${}^{\circ }$	18.61 s
level-6	4.0∼6.0	12.57${}^{\circ }$	17.42 s	1.87${}^{\circ }$	10.02 s	1.63${}^{\circ }$	17.03 s

**Table 2:** The simulation result of monitoring system under traxial sway mode.

Sea Conditions	Bias (${}^{\circ }$/h)			Misalignment Angle (${}^{\circ }$)			Convergence Time (s)
	$\varepsilon_{\mathrm{Fx}}$	$\varepsilon_{\mathrm{Fy}}$	$\varepsilon_{\mathrm{Fz}}$	$\phi_{\mathrm{ax}}$	$\phi_{\mathrm{ay}}$	$\phi_{\mathrm{az}}$	
level-2	0.0954448	0.0996846	0.1014449	1.000	2.000	3.000	74.60
level-4	0.0996374	0.0994061	0.1005486	1.000	2.000	3.000	64.40
level-6	0.0982397	0.0968095	0.0989947	1.000	2.000	3.000	44.80

## Abbreviations

The following abbreviations are used in this manuscript:

CAIG	Cold Atom Interference Gyroscope
FOG	Fiber-optic Gyroscope
INS	Inertial Navigation System
SINS	Strapdown Inertial Navigation System
IMU	Inertial Measurement Unit
PWCS	Piece-wise Constant Systems
